# Effect of potassium deficiency on antioxidant status and cadmium toxicity in rice seedlings

**DOI:** 10.1186/1999-3110-54-2

**Published:** 2013-07-18

**Authors:** Chun-Hsin Liu, Yun-Yang Chao, Ching Huei Kao

**Affiliations:** Department of Agronomy, National Taiwan University, Taipei, Taiwan, ROC

**Keywords:** Antioxidant system, Cadmium, Potassium deficiency, Rice

## Abstract

**Background:**

Cadmium (Cd) is one of the most toxic heavy metals and inhibits physiological processes of plants. Potassium (K) is an essential macronutrient in plants. K deficiency and Cd stress represent two different abiotic stress conditions that occur in the field simultaneously. In this study, effects of K deficiency on antioxidant status and Cd toxicity in rice seedlings were investigated.

**Results:**

K deficiency significantly decreased K concentration in shoots and roots. However, fresh weight and dry weight of rice seedlings were not affected by K deficiency. The activities of antioxidant enzymes (superoxide dismutase, ascorbate peroxidase, glutathione reductase, and catalase) in K-deficient leaves were higher than respective control leaves. However, K deficiency had no effect on the content of antioxidants (ascorbate and glutathione). Cd toxicity was judged by the decrease in biomass production, chlorosis, and induction of oxidative stress. Based on these criteria, we demonstrated that K deficiency protected rice seedling from Cd stress. Moreover, chlorophyll concentration was higher in K-deficient shoots and roots than their respective control shoots and roots.

**Conclusions:**

Our results indicated that K deficiency protects rice seedlings from Cd toxicity. This protective effect of K deficiency is mainly due to enhanced antioxidant enzyme activities but not inhibition of Cd uptake.

**Electronic supplementary material:**

The online version of this article (doi:10.1186/1999-3110-54-2) contains supplementary material, which is available to authorized users.

## Background

Cadmium is a widespread metal contaminating many areas, either naturally or because of industrial use (Pan et al., 2010). Because of its long biological half-life, Cd is highly toxic. In plants, Cd is easily taken up by roots and translocated into shoots. Once Cd is taken up by plants, many cellular structures and metabolic processes are affected (Sanitá di Toppi and Gabbrielli, 1999; Gratǎo et al., 2005). Cd is a bivalent cation and unable to generate free radicals directly, nevertheless the production of reactive oxygen species (ROS) after Cd exposure has been reported (Sanitá di Toppi and Gabbrielli, 1999; Gratǎo et al., 2005; Cuypers et al., 2010). ROS has to be kept under tight control because their accumulation results in cell death due to oxidative processes such as lipid peroxidation, protein oxidation, and DNA damage (Gill and Tuteja, 2010). Plants use antioxidant enzymes such as superoxide dismutase (SOD), ascorbate peroxidase (APX), glutathione reductase (GR), and catalase (CAT) as well as non-enzymatic antioxidants such as ascorbate (AsA) and glutathione (GSH) to scavenge ROS (Noctor and Foyer 1998; Amudha and Balasubnramani 2011; Foyer and Noctor, 2011).

In agricultural systems, high-density monoculture crops deplete soil minerals quickly and therefore rely on external supplies for most of their nutrients, particularly nitrogen, potassium (K) and phosphorus (Amtmann et al., 2006). A balanced supply of mineral nutrients is crucial for both quantity and quality of the crop, but rarely achieved in the field. Of the mineral nutrients, imbalanced nutrition with K is well known and becoming an important constraint to crop production in many areas (Cakmak, 2010).

Potassium (K) is an essential macronutrient and plays an important role in metabolism as it functions as a cofactor of many enzymes and is required for charge balance and transport of metabolites (Marschner, 1995). It has been shown that the rate of net photosynthesis and the activity of ribulose-1, 5-bisphosphate carboxylase decrease in plants under conditions of K deficiency (Peoples and Koch 1979; Zhao et al., 2001; Cakmak, 2005; Weng et al., 2007). Several lines of evidence have also shown that K deficiency causes a decrease in sucrose export from source leaves (Mengel and Viro, 1974; Cakmak, 2005). Thus, the impairment in photosynthetic CO_2_ fixation and decrease in sucrose export in K-deficient leaves could lead to enhanced oxygen photoreduction in the chloroplast via the Mehler reaction resulting in the production of ROS. In order to detoxify ROS, increases in the activities/contents of antioxidants are expected in leaves of K-deficient plants. Indeed, enhancement in the activities of antioxidant enzymes has been demonstrated in K-deficient bean leaves (Cakmak, 1994,2005). Ding et al. (2008) also reported that the activities of SOD, CAT and peroxidase in the leaves of rice plants supplied with low K (0.5 mM) were higher than those supplied with high K (6 mM).

In the field, crops and other plants are routinely subjected to a combination of different abiotic stresses. For this reason, the importance of focusing the research programs in the response of plants to a combination of two different abiotic stresses has been emphasized (Mittler, 2006). Recently, we have revealed that Mg deficiency protects rice seedlings from Cd stress (Chou et al., 2011). In contrast, N deficiency was observed to enhance subsequent toxicity in rice seedlings caused by Cd stress (Lin et al., 2011). K deprivation and Cd stress represent another example of two different abiotic stress conditions that occur in the field simultaneously. However, it is not known whether Cd stress of rice seedlings is influenced by K deficiency. The present study was undertaken with the objective to examine the effect of K deficiency on antioxidant status and subsequent Cd-induced toxicity in rice seedlings.

## Material and methods

### Plant material and growth conditions

Rice (*Oryza sativa* L., cv. Taichung Native 1) seeds were sterilized with 2.5% sodium hypochlorite for 15 min and washed extensively with distilled water. These seeds were then germinated in Petri dishes with wetted filter papers at 37°C in the dark. After 48 h incubation, uniformly germinated seeds were selected and cultivated in a beaker containing half-strength Kimura B nutrient solution with sufficient K supply (control) or deficient K supply (−K). Nutrient solution for the control contains the following macro- and micro-elements: 182.3 μM (NH_4_)_2_SO_4_, 91.6 μM KNO_3_, 273.9 μM MgSO_4_, 7H_2_O, 91.1 μM KH_2_PO_4_, 182.5 μM Ca(NO_3_)_2_, 30.6 μM Fe-citrate, 0.25 μM H_3_BO_3_, 0.2 μM MnSO_4_ · H_2_O, 0.2 μM ZnSO_4_ · 7H_2_O, 0.05 μM CuSO_4_ · 5H_2_O and 0.07 μM H_2_MoO_4_ (Kimura, 1931). Sodium nitrate substituted for KNO_3_ and NaH_2_PO_4_ for KH_2_PO_4_, respectively, under K-deficient conditions. Kimura B nutrient solution contains the desired nutrients for growing rice plants. Since young rice seedlings were used for the present study, the nutrient solution contained no silicon, although silicon is essential for growth of sturdy rice plants in the field.

Nutrient solution (pH 4.7) were replaced every 3 days. The hydroponically cultivated seedlings were grown in a Phytotron (Agricultural Experimental Station, National Taiwan University, Taipei, Taiwan) with natural sunlight at 30/25°C day/night and 90% relative humidity. Four beakers were used for each treatment, with 20 seedlings in each beaker. Twelve-day-old seedlings with three leaves were then grown with or without CdCl_2_ (5 μM) for 6 days. Cd toxicity (chlorosis, chlorophyll loss, and lipid peroxidation) was first shown in the second leaves of rice seedlings (Hsu and Kao, 2005). For this reason, unless otherwise indicated the second leaves of rice seedlings were used to perform all the chemical measurements and enzyme assays.

### Determination of K and Cd

For determination of K and Cd, samples were dried at 65°C for 2 days. Dried material was ashed at 550°C for 4 days. The ash residue was incubated with 70% HNO_3_ and 30% H_2_O_2_ at 72°C for 2 h, and dissolved in distilled water for detection of K and Cd. K and Cd concentrations were then quantified using an atomic absorption spectrophotometer (Model AA-6800, Shimadzu, Kyoto, Japan) and expressed on a dry weight (DW) basis.

### Growth response

At the end of treatment, the seedlings were divided into shoots and roots. For DW estimation, the shoots and roots were dried at 65°C for 48 h, by which time the DW remains constant.

### Determination of H_2_O_2_, chlorophyll, and MDA

The H_2_O_2_ was extracted with sodium phosphate buffer (50 mM, pH 6.8) containing 1 mM hydroxylamine, a catalase inhibitor. The H_2_O_2_ content was measured after reaction with TiCl_4_ (Tsai et al., 2004). The blank reaction consisted of 50 mM phosphate buffer in the absence of leaf extracts. The absorbance was measured at 410 nm. The amount of H_2_O_2_ was calculated by using a standard curve prepared with known concentrations of H_2_O_2_.

The chlorophyll content was determined according to Wintermans and De Mots (1965) after extraction in 96% (v/v) ethanol. For protein determination, leaves were homogenized in a 50 mM sodium phosphate buffer (pH 6.8). The extracts were centrifuged at 17,600 *g* for 20 min, and the supernatants were used for determination of protein by the method of Bradford (1976). Malondialdehyde (MDA), routinely used as an indicator of lipid peroxidation, was extracted with 5% (w/v) trichloroacetic acid and determined by the thiobarbituric acid reaction as described by Heath and Packer (1968). The contents of H_2_O_2_, chlorophyll, and MDA were expressed on the basis of fresh weight (FW) of the second leaves prior to Cd treatment.

### Determination of AsA, dehydroascorbate (DHA), GSH, andoxidized glutathione (GSSG)

Ascorbic acid (AsA) and DHA contents in 5% (w/v) trichloroacetic acid were determined as described by Law et al. (1983). The assay is based on the reduction of Fe^3+^ to Fe^2+^ by AsA. The Fe^2+^ then forms complexes with bipyridyl, giving a pink color that absorbs at 525 nm. GSH and GSSG contents in 3% sulfosalicylic acid extract were determined by the method of Smith (1985). The content of GSH was spectrophotometrically determined with an enzyme-recycling assay at 412 nm. The assay is based on sequential oxidation of GSH by 5, 5-dithiobis-(2-nitrobenzoic acid) and reduction by NADPH in the presence of known amount of GR. The contents of AsA, DHA, GSH and GSSG were expressed on the basis of FW of the second leaves prior to Cd treatment.

### Enzyme extraction and assays

Leaf samples were excised and immediately used for enzyme extraction. All operations were carried out at 4°C. For extraction of enzymes, leaf tissues (about 70 mg FW) were homogenized with 0.1 M sodium phosphate buffer (pH 6.8) in a chilled pestle and mortar. For analysis of APX activity, 2 mM AsA was added to the extraction buffer. The homogenate was centrifuged at 12,000 *g*. SOD activity was determined according to Paoletti et al. (1986). This method, originally used for animal material (liver), has been used to determine SOD activity in rice (Dey and Kar, 1995), maize (Bennicelli et al., 1998), cucumber (Piacentini et al., 2001), wheat (Goggin and Colmer, 2005), and potato (Agrawal et al., 2008). The reaction mixture (2.73 mL) contained 100 mM triethanolamine-diethanolamine buffer (pH 7.4), 7.5 mM NADH, EDTA/MnCl_2_ (100 mM/50 mM, pH 7.4), 10 mM 2-mercaptoethanol, and enzyme extract (0.2 mL). The reaction was started by the addition of NADH. The reaction was allowed to proceed for 10 min. The absorbance was measured at 340 nm. One unit of SOD was defined as the amount of enzyme that inhibited by 50% the rate of NADH oxidation observed in blank sample. CAT activity was assayed according to Kato and Shimizu (1987). The decrease in H_2_O_2_ was followed as the decline in the absorbance at 240 nm, and the activity was calculated using the extinction coefficient (40 mM^-1^ cm^-1^ at 240 nm) for H_2_O_2_. One unit of CAT was defined as the amount of enzyme which degraded 1 μmol H_2_O_2_ per min. APX activity was determined according to Nakano and Asada (1981). The decrease in AsA concentration was followed as a decline in the absorbance at 290 nm and activity was calculated using the extinction coefficient (2.8 mM^-1^ cm^-1^ at 290 nm) for AsA. One unit of activity for APX was defined as the amount of enzyme that degraded 1 μmol of AsA per min. GR was defined as the amount of enzyme of Foster and Hess (1980). One unit of GR was defined as the amount of enzyme that decreased 1 optical density min^-1^ at 340 nm. Enzyme activities were expressed on the basis of mg protein.

### Paraquat resistance

To test if the protection effect of K deficiency against Cd toxicity is due to enhanced protection against oxidative stress, the second leaves were detached from 12-day-old rice seedlings grown under K-sufficient and -deficient nutrition solutions. Ten detached leaves were then floated on Petri dish containing 10 μM paraquat (PQ) at 27°C for 24 h in the light (40 μmol m^-2^ s^-1^). After incubation, 10 detached leaves per treatment (four replicates) were analyzed for chlorophyll.

### Statistical analyses

Data were analyzed by Student’s *t*-test or Duncan’s multiple range test. *P* < 0.05 was considered statistically significant.

## Results

### Effect of K deficiency on growth response and K concentration

**Figure 1:**
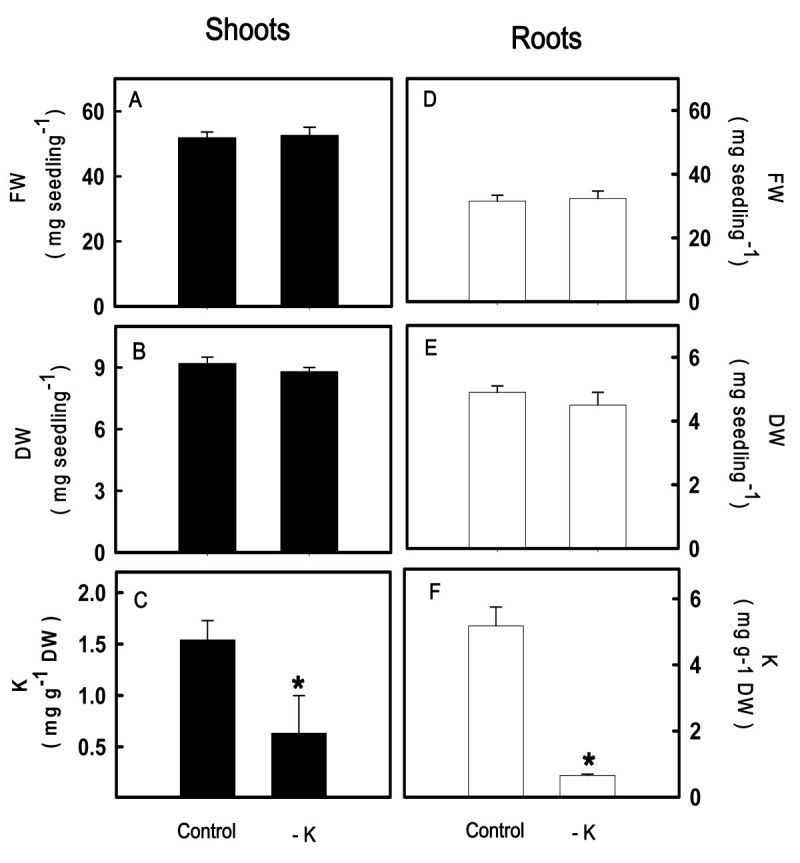
**The fresh weight (FW) (A, D), dry weight (DW) (B, E) and K concentration (C, F) in shoots and roots of rice seedlings.** Rice seedlings were grown under K-sufficient (control) and -deficient (−K) conditions for 12 days. The shoots and roots were then used to determine FW, DW, and K concentration. Bars indicate standard errors (*n* = 4). Asterisks represent values that are significantly different between control and − K treatments at *P* < 0.05.

To examine the effect of K deficiency on growth response and K concentration, rice seedlings were grown under conditions of sufficient (control) and deficient K supply (−K) for 12 days. The FW and DW of both shoots and roots were not affected by K deficiency (Figure 1A, B, D, E). However, K deficiency resulted in a decrease in K concentration in shoots and roots (Figure 1C, F).

### Effect of K deficiency on antioxidant status

**Figure 2:**
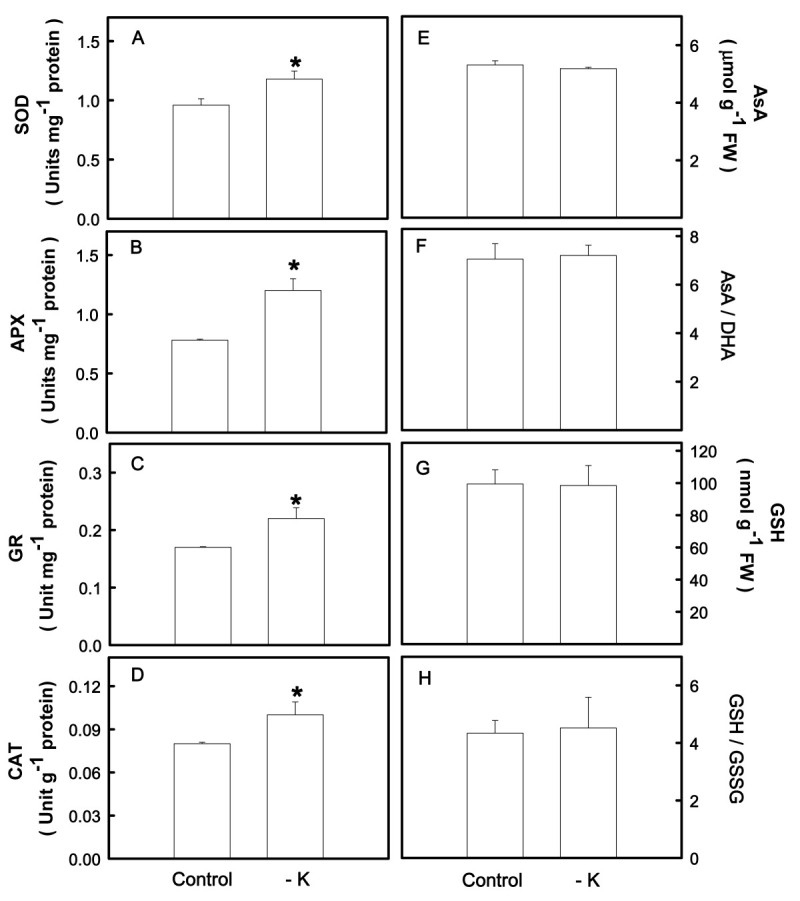
**The activities of superoxide dismutase (SOD) (A), ascorbate peroxidase (APX) (B), glutathione reductase (GR) (C), and catalase (CAT) (D) and the contents of ascorbate (AsA) (E) and reduced glutathione (GSH) (G) and the ratios of AsA/dehydroascorbate (DHA) (F) and GSH/oxidized glutathione (GSSG) (H) in the second leaves.** Rice seedlings were grown under K-sufficient (control) and -deficient (−K) conditions for 12 days. The second leaves were then used to determine the contents of AsA, DHA, GSH, and GSSG. Bars indicate standard errors (*n* = 4). Asterisks represent values that are significantly different between control and − K treatments at *P* < 0.05.

Effects of K deficiency on the activities of antioxidant enzymes in the second leaves were shown in Figure 2A-D. K deficiency significantly increased the activities of SOD, APX, GR, and CAT in the second leaves. In the present study, the effect of K deficiency on the contents of antioxidants in the second leaves was also investigated. K deficiency had no effect on the contents of AsA and GSH and the ratios of AsA/DHA and GSH/GSSG in the second leaves (Figure 2E-H).

### Effect of K deficiency on Cd-induced changes in biomass production

**Figure 3:**
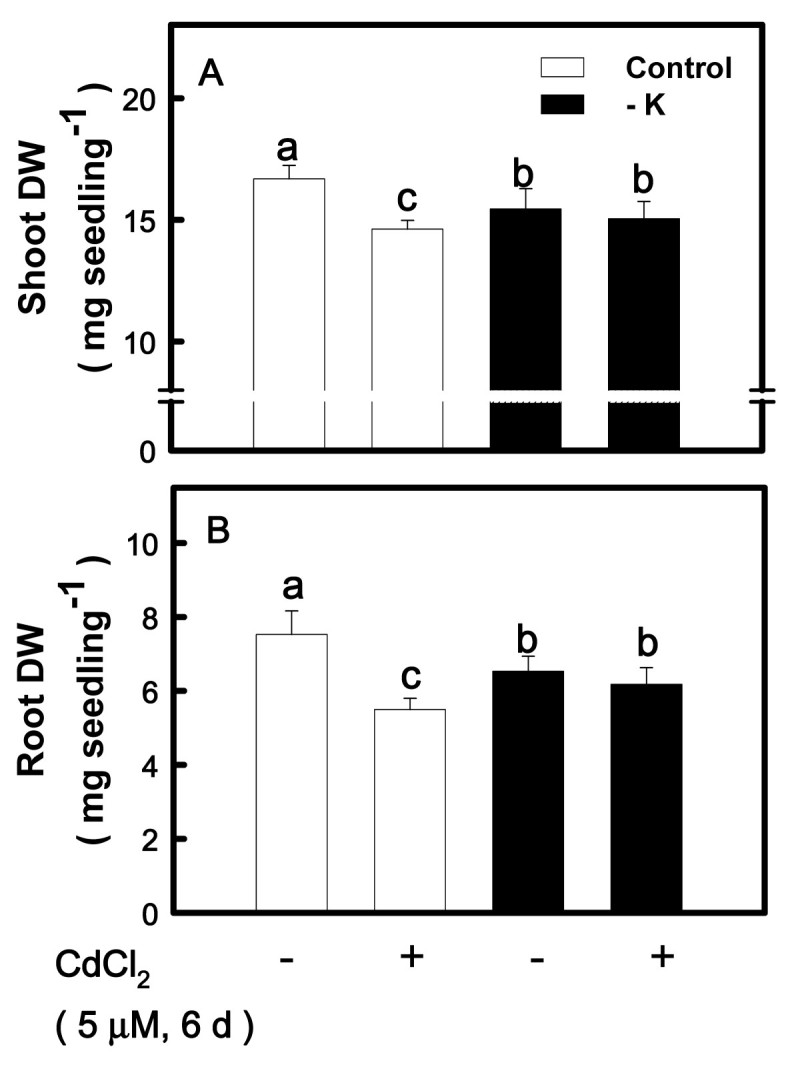
**Effect of CdCl**_**2**_**on the DW of shoots (A) and roots (B) of rice seedlings grown under K-sufficient (control) and -deficient (−K) conditions.** Rice seedlings were grown under control and − K conditions for 12 days. Control and − K seedlings were then transferred to the control and − K nutrient solution with or without 5 μM CdCl_2_ for 6 days, respectively. The shoots and roots were then used to determine DW. Bars indicate standard errors (*n* = 4). Values with the same letter are not significantly different at *P* < 0.05.

Cd is readily taken up by rice seedlings, leading to growth reduction (Chen and Kao, 1995). Thus, in the present study, Cd toxicity was first evaluated by biomass production (shoot and root DW). The DW of the shoots and roots in K-sufficient seedlings was significantly decreased by Cd (Figure 3A, B). However, Cd had no effect on the DW of the shoots and roots in − K seedlings (Figure 3A, B).

### Effect of K deficiency on Cd-induced chlorosis

**Figure 4:**
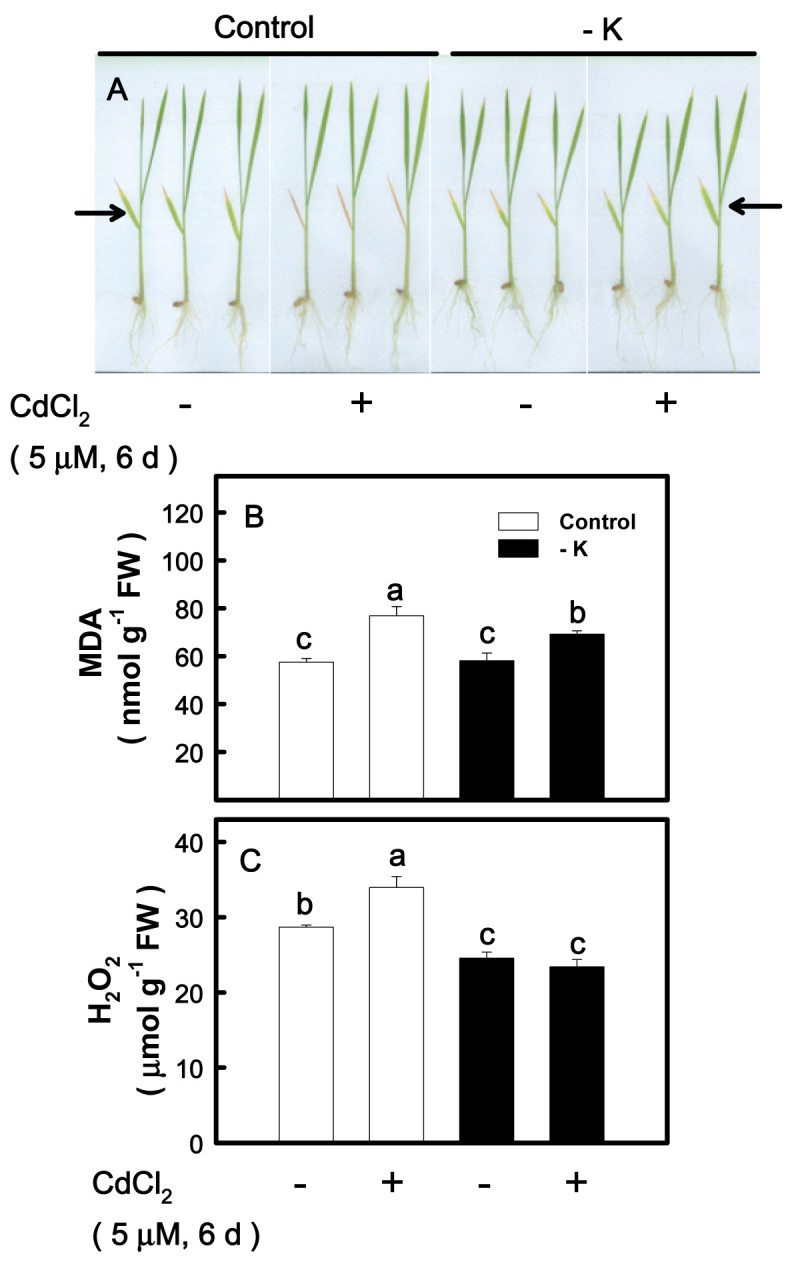
**Effect of CdCl**_**2**_**on the chlorosis (A) and the contents of malondialdehyde (MDA) (B), and H**_**2**_**O**_**2**_**(C) in the second leaves of rice seedlings grown under K-sufficient (control) and -deficient (−K) conditions.** Rice seedlings were grown under control and − K conditions for 12 days. Control and − K seedlings were then transferred to the control and − K nutrient solution with or without 5 μM CdCl_2_ for 6 days, respectively. The second leaves were then used to determine the chlorosis and contents of MDA and H_2_O_2_. Arrows indicate the second leaves. Bars indicate standard errors (*n* = 4). Values with the same letter are not significantly different at *P* < 0.05.

In plants, the general symptom of Cd toxicity is chlorosis (Das et al., 1997). When rice seedlings were treated with CdCl_2_, chlorosis was first shown in the second leaves (Hsu and Kao, 2005). Thus, the second leaves were used to determine chlorosis in the present study. To test if K deficiency would affect Cd stress-induced chlorosis, 12-day-old control and K-deficient seedlings were transferred to K-sufficient and -deficient nutrient solution with or without 5 μM CdCl_2_ for 6 days, respectively. Cd-induced chlorosis in K-deficient leaves was less pronounced than their respective control leaves (Figure 4A).

### Effect of K deficiency on Cd-induced oxidative stress

Previous work demonstrated that Cd induces oxidative stress in rice leaves, characterized by an increase in the contents of MDA (an indicator of lipid peroxidation) and H_2_O_2_ (Kuo and Kao, 2004; Hsu and Kao, 2007). It was observed that the increase in the contents of MDA and H_2_O_2_ caused by 5 μM CdCl_2_ was less pronounced in K-deficient leaves than in control leaves (Figure 4B, C).

**Figure 5:**
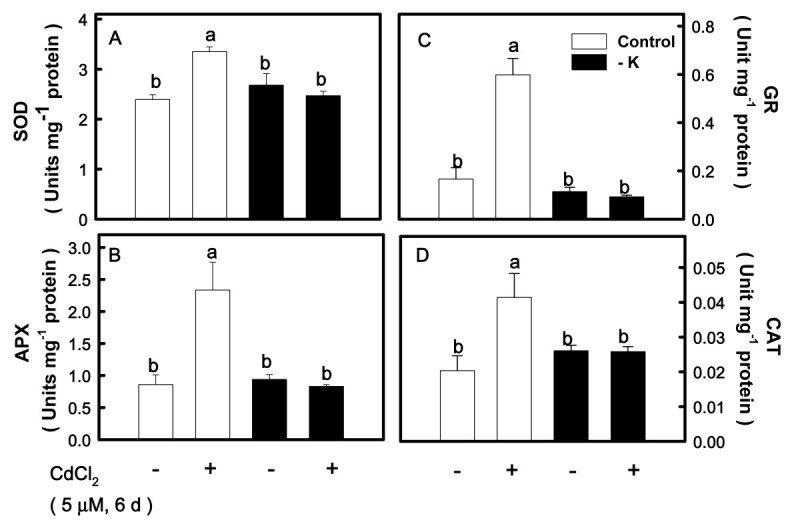
**Effect of CdCl**_**2**_**on the activities of SOD (A), APX (B), GR (C) and CAT (D) in the second leaves of rice seedlings grown under K-sufficient (control) and -deficient (−K) conditions.** Rice seedlings were grown under control and − K conditions for 12 days. Control and − K seedlings were then transferred to the control and − K nutrient solution with or without 5 μM CdCl_2_ for 6 days, respectively. The second leaves were then used to determine enzyme activities. Bars indicate standard errors (*n* = 4). Values with the same letter are not significantly different at *P* < 0.05.

**Figure 6:**
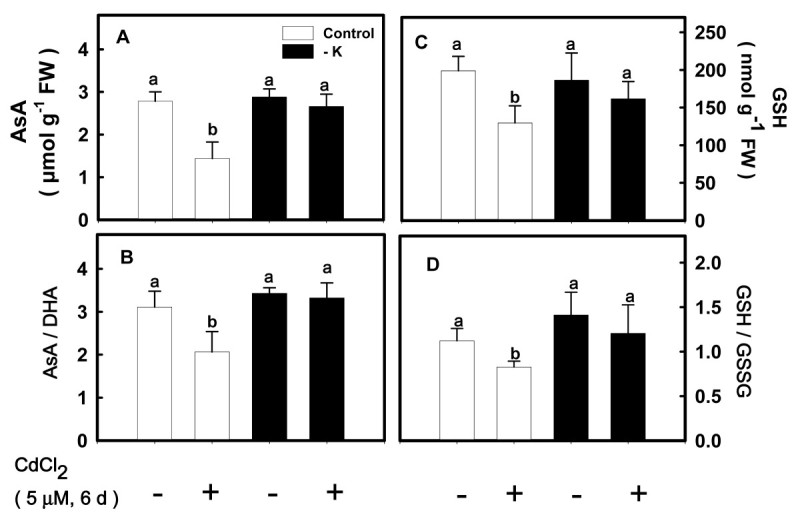
**Effect of CdCl**_**2**_**on the contents of AsA (A) and GSH (C) and the ratios of AsA/DHA (B) and GSH/GSSG (D) in the second leaves of rice seedlings grown under K-sufficient (control) and -deficient (−K) conditions.** Rice seedlings were grown under control and − K conditions for 12 days. Control and − K seedlings were then transferred to the control and − K nutrient solution with or without 5 μM CdCl_2_ for 6 days, respectively. The second leaves were then used to determine the contents of AsA, DHA, GSH, and GSSG. Bars indicates standard errors (*n* = 4). Values with the same letter are not significantly different at *P* < 0.05.

The striking increase in lipid peroxidation and H_2_O_2_ seen in K-sufficient leaves treated with CdCl_2_ (Figure 4B, C), may reflect changes in the specific activities of antioxidant enzymes and contents of antioxidants. Here, we showed that the increase in the activities of SOD, APX, GR, and CAT caused by Cd was more pronounced in K-sufficient leaves than in K-deficient leaves (Figure 5A-D). AsA and GSH are important antioxidants in plants (Gill and Tuteja, 2010; Cuypers et al., 2010). It was observed that the decrease in ASA and GSH contents caused by CdCl_2_ was greater in K-sufficient leaves than in K-deficient leaves. The results of this study also demonstrated that Cd treatment resulted in a significant decrease in the AsA/DHA and GSH/GSSG ratios in K-sufficient leaves (Figure 6B, D). However, the AsA/DHA and GSH/GSSG ratios in − K leaves were not affected by CdCl_2_ (Figure 6B, D).

### Effect of K deficiency on paraquat-induced chlorophyll loss

**Figure 7:**
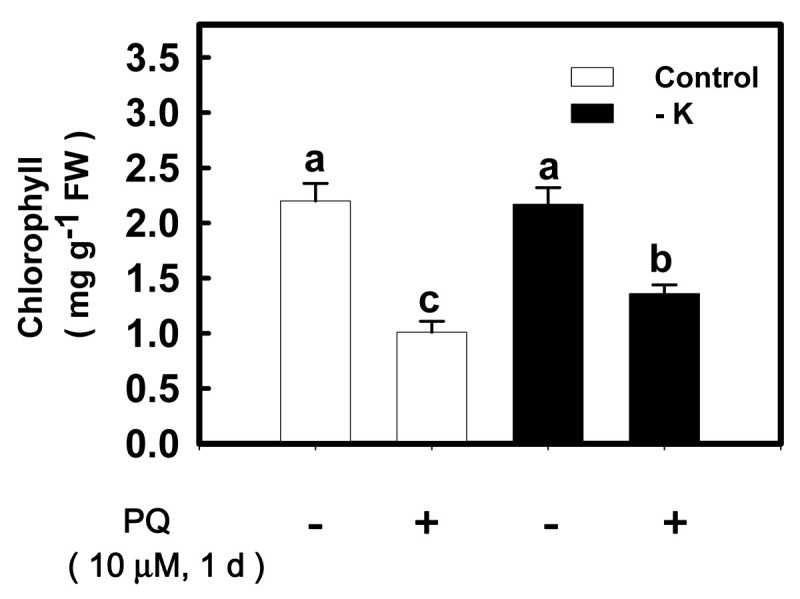
**Effect of paraquat (PQ) on chlorophyll content of detached leaves from K-sufficient (control) and -deficient (−K) conditions.** Rice seedlings were grown under control and − K conditions for 12 days. The second leaves were excised from control and − K seedlings and then transferred to Petri dishes containing H_2_O and 10 μM PQ, respectively, for 24 h under light conditions. Detached leaves were then used to determine chlorophyll content. Bars indicate standard errors (*n* = 4). Values with the same letter are not significantly different at *P* < 0.05.

Irrespective of the K supply, floating detached leaves in 10 μM PQ solution caused a decrease in chlorophyll content (Figure 7). The decrease in chlorophyll content by PQ became more evident in K-sufficient compared with K-deficient leaves (Figure 7).

### Effect of K deficiency on the concentration of Cd

**Figure 8:**
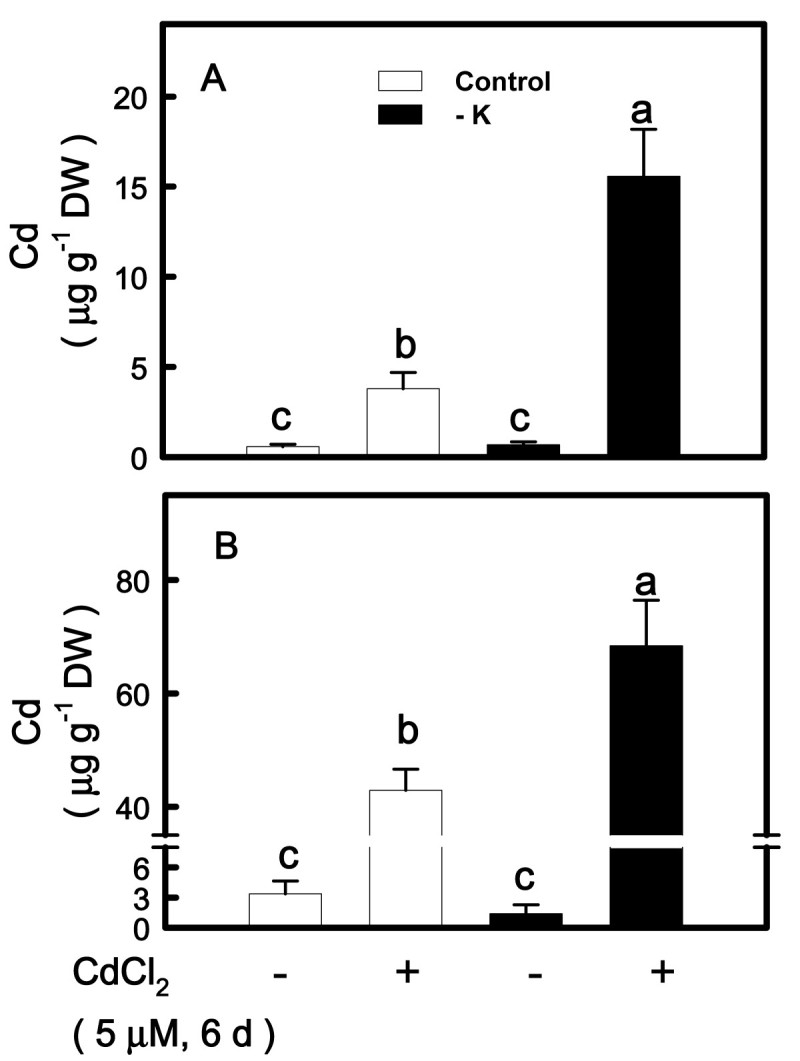
**Effect of CdCl**_**2**_**on the concentrations of Cd in shoots (A) and roots (B) of rice seedlings grown under K-sufficient (control) and -deficient (−K) conditions.** Rice seedlings were grown under control and − K conditions for 12 days. Control and − K seedlings were then transferred to the control and − K nutrient solution with or without 5 μM CdCl_2_ for 6 days, respectively. The shoots and roots were then used to determine Cd concentration. Bars indicate standard errors (*n* = 4). Values with the same letter are not significantly different at *P* < 0.05.

To test if K deficiency would affect the uptake of Cd, 12-day-old control and K-deficient seedlings were transferred to K-sufficient and -deficient nutrient solutions with or without 5 μM CdCl_2_ for 6 days, respectively. Contrary to our expectation, It was observed that shoots and roots of K-deficient seedlings had higher Cd concentration than those of K-sufficient seedlings (Figure 8A, B). Irrespective of the K supply, Cd concentration in roots (Figure 8B) was significantly higher than that in shoots (Figure 8A).

## Discussion

In the present study, rice seedlings were grown under K-sufficient and -deficient conditions for 12 days, by which time the third leaves of both K-sufficient and -deficient rice seedlings were fully grown. K deficiency significantly decreased K concentrations in shoots and roots of rice seedlings (Figure 1C, F). However, neither DW nor FW in shoots and roots was affected by K deficiency (Figure 1A, B, D, E). These results suggest that plant biomass is not effective for diagnosis of K deficiency in the early stage. It appears that K analysis rather than plant biomass is the most accurate tool to diagnose K deficiency.

In this study, Na salt substituted for K salt under K-deficient conditions. Elimination of K in this study by Na gives not only elimination of K but also leads to increase in Na concentration. It has been shown that NaCl inhibited growth of rice seedlings (Lin and Kao, 1995). Since growth of rice seedlings was not affected by K deficiency (Figure 1A, B, D, E), thus, Na taken up by rice seedlings does not seem to raise the concentration of the Na in the tissue to the levels that are toxic to rice growth under K deficiency conditions.

In the present study, Cd toxicity was evaluated by the decrease in biomass production, chlorosis, and induction of oxidative stress. On the basis of these criteria, it was demonstrated that K deficiency protected rice seedlings from the toxicity caused by 5 μM CdCl_2_ (Figures 3–6). PQ is an herbicide widely used in agriculture and a well known ROS-generating chemical. Treatment of detached rice leaves with 10 μM PQ for 24 h in the light caused marked chlorophyll loss. This chlorophyll destruction caused by PQ was higher in K-sufficient than K-deficient leaves (Figure 7). Clearly, K deficiency is able to protect rice seedlings from ROS damage caused by Cd or PQ. Thus, the K nutritional status of plants should be taken into consideration not only in evaluation of Cd toxicity but also in screening studies for PQ resistance. It is well known that PQ resistant plants also posses a higher resistance to O_3_ and SO_2_ (Shaalitiel et al., 1988; Tanaka et al., 1988). Based on the data shown in this study, it might be assumed that K-deficient leaf tissues are also more resistant to other ROS activating stress factors such as O_3_ and SO_2_.

It has been established that the increase in the activities of antioxidant enzymes and /or the contents of antioxidants is important for the improvement of stress tolerance (Gill and Tuteja, 2010). Cho and Seo (2005) demonstrated that seedlings of Cd-resistant *Arabidopsis* had higher activities of SOD, APX and GR and experienced lower oxidative stress from Cd exposure. The results of this study show that K-deficient rice leaves have higher activities of antioxidant enzymes, SOD, APX, GR, and CAT than K-sufficient leaves (Figure 2A-D). Enhanced activities of antioxidant enzymes have also been described in K-deficient bean leaves (Cakmak, 1994) and rice plants (Ding et al., 2008).

Irrespective of K supply, Cd treatment resulted in an increase in Cd concentration in rice roots and shoots (Figure 8A, B). The Cd concentration was lower in shoots than in roots (Figure 8A, B), indicating that a higher proportion of the Cd taken up by rice remained in the roots. This is in agreement with previous reports (Jalil et al., 1994; Wu and Zhang, 2002; Ueno et al., 2010). Figure 8 showed that Cd concentration was higher in K-deficient shoots and roots than their respective control shoots and roots. Thus, the protective effect of K deficiency against Cd toxicity is mainly due to enhanced antioxidant status but not inhibition of Cd uptake. It appears that K-deficient leaves experienced lower oxidative stress. In this study, the total shoot and root Cd concentrations were measured. It is not known whether K deficiency alters Cd distribution between vacuolar compartment and the rest of the cell. In future studies, it will be important to determine the effect of K deficiency on Cd concentration in different cellular compartments.

## Conclusion

In conclusion, our results indicated that K deficiency protects rice seedlings from Cd toxicity. This protective effect of K deficiency is mainly due to enhanced antioxidant enzyme activities but not inhibition of Cd uptake.

## Authors’ original submitted files for images

Below are the links to the authors’ original submitted files for images. Authors’ original file for figure 1 Authors’ original file for figure 2 Authors’ original file for figure 3 Authors’ original file for figure 4 Authors’ original file for figure 5 Authors’ original file for figure 6 Authors’ original file for figure 7 Authors’ original file for figure 8

## Abbreviations

APX: Ascorbate peroxidase
AsA: Ascorbic acid
CAT: Catalase
DHA: Dehydroascorbate
DW: Dry weight
FW: Fresh weight
GR: Glutathione reductase
GSH: Reduced glutathione
GSSG: Oxidizdd glutathione
PQ: Paraquat
ROS: Reactive oxygen species
MDA: Malondialdehyde
SOD: Superoxide dismutase.
