# Supplementary figures and images for: Effect of potassium deficiency on antioxidant status and cadmium toxicity in rice seedlings
Source: Bot Stud. 2013 Jul 18;54:2. doi: 10.1186/1999-3110-54-2 (PMC5383923; doi:10.1186/1999-3110-54-2)

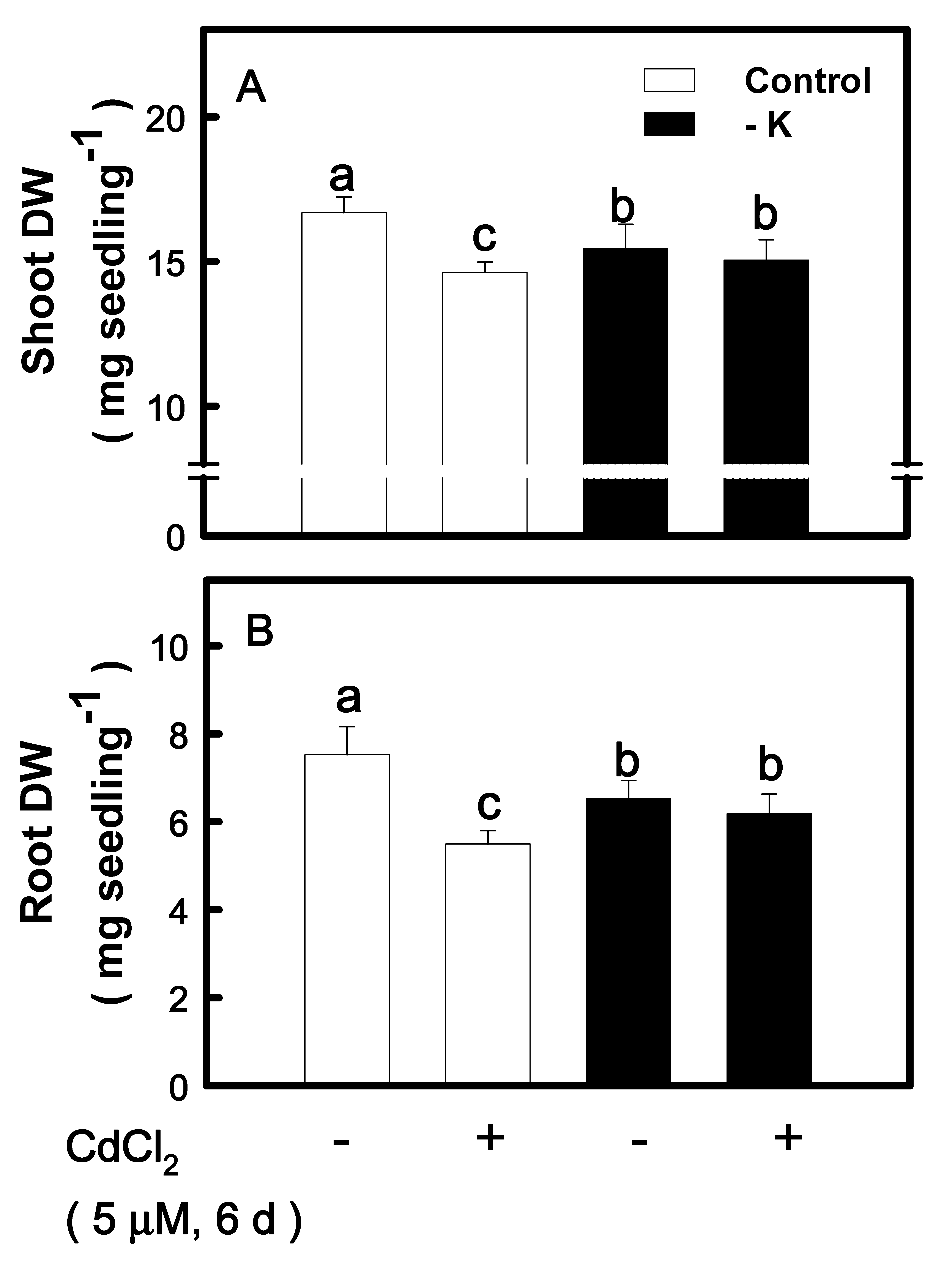

Supplement: Supplementary file 3 — Authors’ original file for figure 3 [file 40529_2013_1_MOESM3_ESM.tiff]

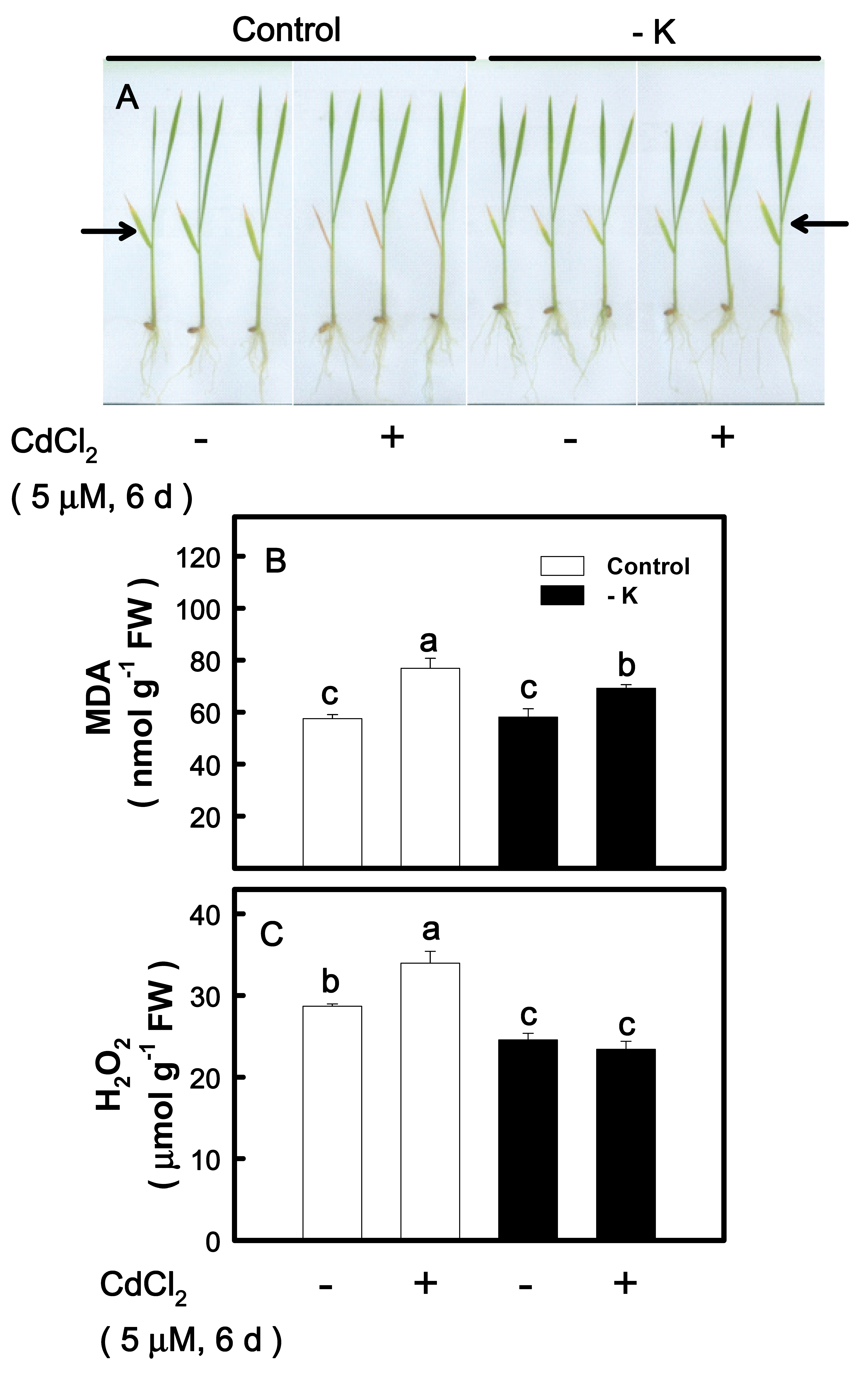

Supplement: Supplementary file 4 — Authors’ original file for figure 4 [file 40529_2013_1_MOESM4_ESM.tiff]

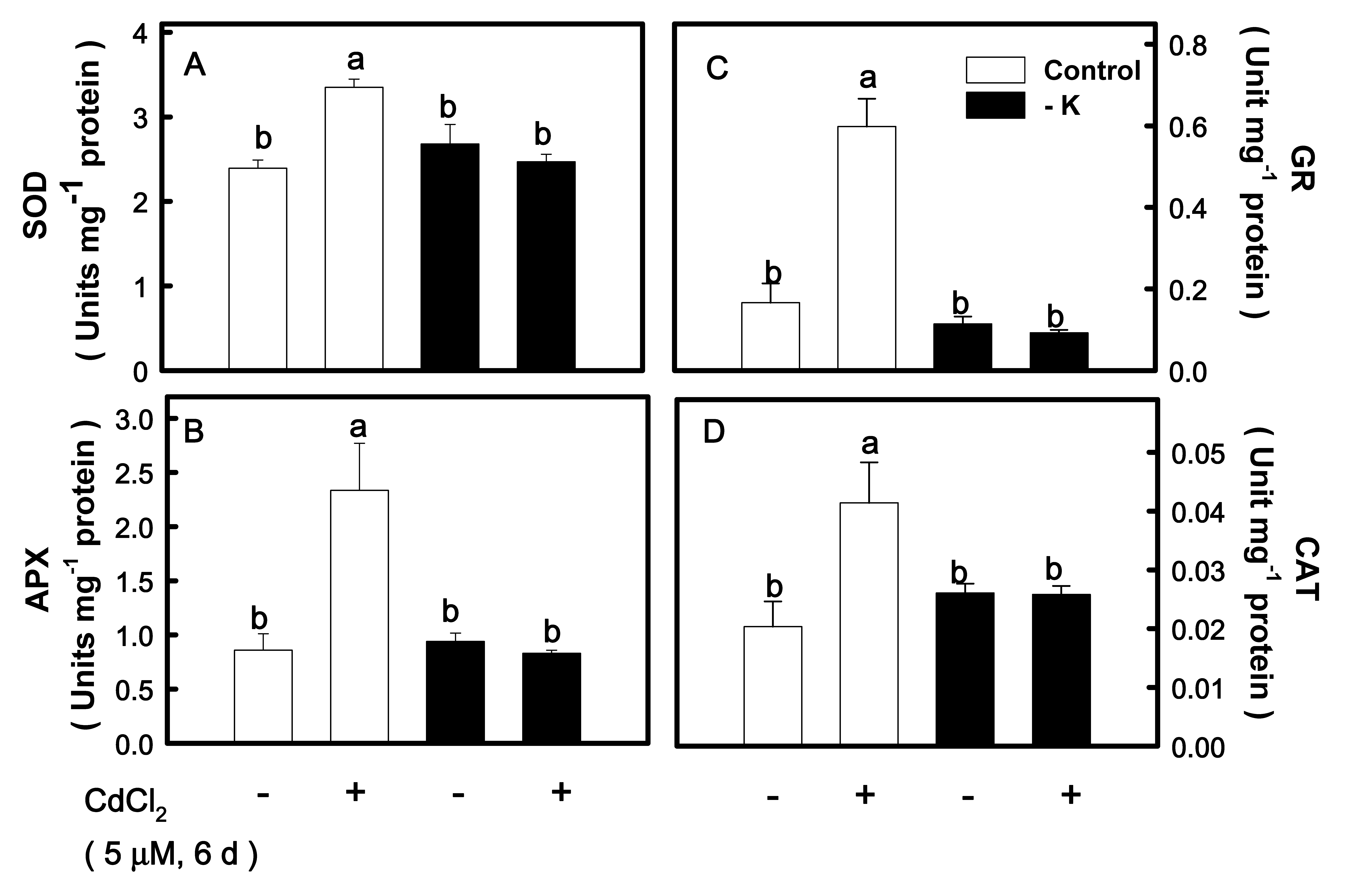

Supplement: Supplementary file 5 — Authors’ original file for figure 5 [file 40529_2013_1_MOESM5_ESM.tiff]

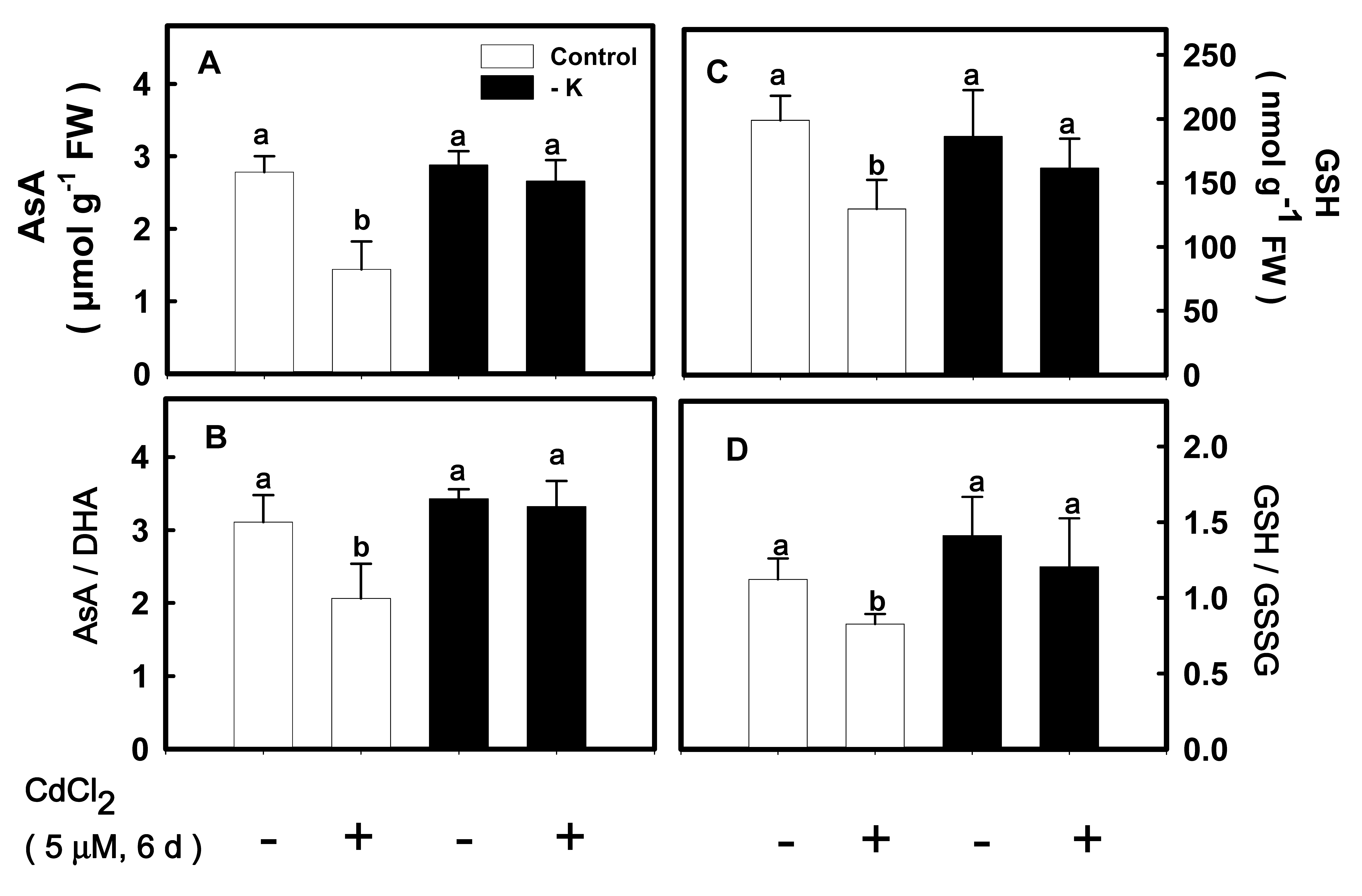

Supplement: Supplementary file 6 — Authors’ original file for figure 6 [file 40529_2013_1_MOESM6_ESM.tiff]

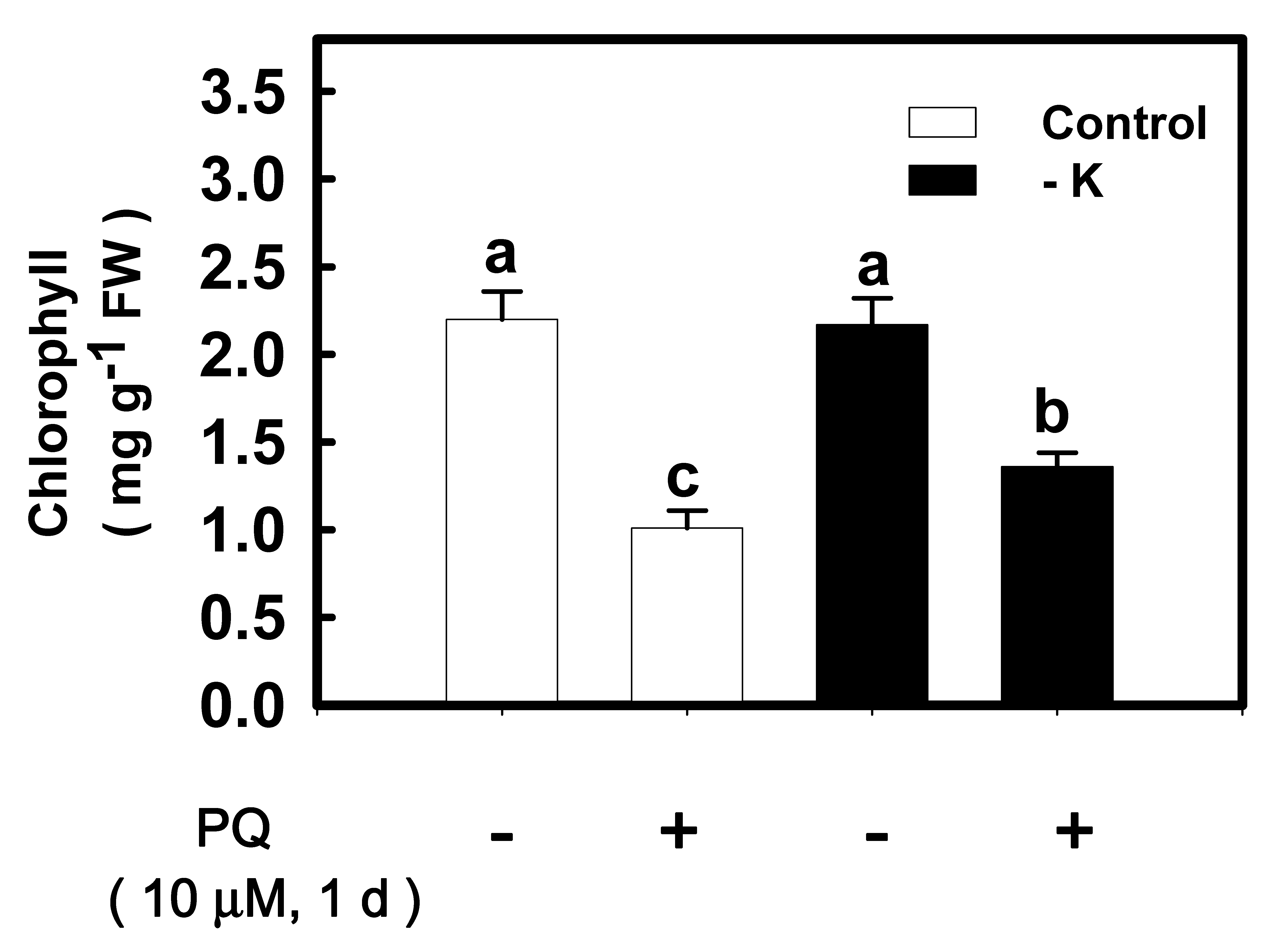

Supplement: Supplementary file 7 — Authors’ original file for figure 7 [file 40529_2013_1_MOESM7_ESM.tiff]

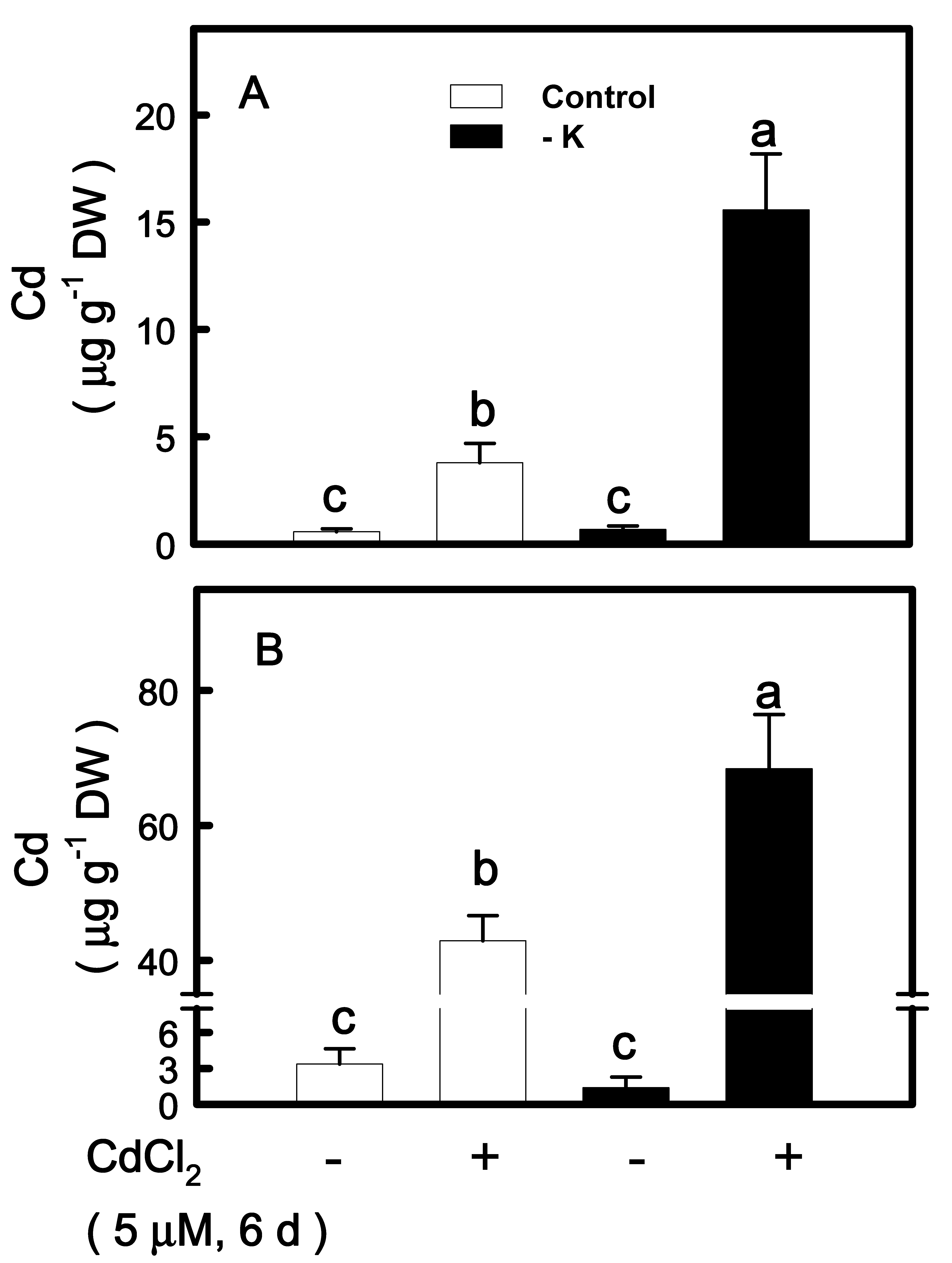

Supplement: Supplementary file 8 — Authors’ original file for figure 8 [file 40529_2013_1_MOESM8_ESM.tiff]
